# HPV16 E5 deregulates the autophagic process in human keratinocytes

**DOI:** 10.18632/oncotarget.3326

**Published:** 2015-03-19

**Authors:** Francesca Belleudi, Monica Nanni, Salvatore Raffa, Maria Rosaria Torrisi

**Affiliations:** ^1^ Istituto Pasteur-Fondazione Cenci Bolognetti, Dipartimento di Medicina Clinica e Molecolare, Sapienza Università di Roma, Rome, Italy; ^2^ Azienda Ospedaliera S. Andrea, Rome, Italy

**Keywords:** HPV, KGF/FGF7, KGFR, FGFR2b, autophagy

## Abstract

Autophagy plays key roles during host defense against pathogens, but viruses have evolved strategies to block the process or to exploit it for replication and successful infection. The E5 oncoprotein of human papillomavirus type 16 (HPV16 E5) perturbs epithelial homeostasis down-regulating the expression of the keratinocyte growth factor receptor (KGFR/FGFR2b), whose signaling induces autophagy. Here we investigated the possible effects of 16E5 on autophagy in human keratinocytes expressing the viral protein. The 16E5 presence strongly inhibited the autophagic process, while forced expression and activation of KGFR counteracted this effect, demonstrating that the viral protein and the receptor exert opposite and interplaying roles not only on epithelial differentiation, but also in the control of autophagy. In W12 cells, silencing of the 16E5 gene in the context of the viral full length genome confirmed its role on autophagy inhibition. Finally, molecular approaches showed that the viral protein interferes with the transcriptional regulation of autophagy also through the impairment of p53 function, indicating that 16E5 uses parallel mechanisms for autophagy impairment. Overall our results further support the hypothesis that a transcriptional crosstalk among 16E5 and KGFR might be the crucial molecular driver of epithelial deregulation during early steps of HPV infection and transformation.

## INTRODUCTION

Autophagy is a highly regulated “self-digestion” pathway [1], which is enhanced by cellular stresses, such as nutrient starvation or hypoxia [2], and plays a crucial role during host defense, permitting pathogens detection and their rapid lysosomal degradation [3]. Recently, a close interplay between autophagy and oncogenic virus infection has been proposed: in fact, while host cells use the autophagosome assembly for virus particle isolation and their sorting to the lysosomal pathway, many viruses have evolved strategies to block the autophagic flux or to exploit the process in order to improve their replication [4, 5].

The infection with high-risk genotypes of human papillomaviruses (HPVs), such as the human papillomavirus type 16 (HPV16) and type 18 (HPV18), represents a major risk factor for cervical cancer development and progression [6, 7]. It has been recently shown that HPV16 infection induces autophagy in host epithelial cells and the virus infectivity is strongly affected by the extent of the autophagic response [8]. On the other hand, the depletion of all the HPV16 early proteins resulted in a strong increase of autophagy in infected cervical keratinocytes [9]. Even if this latter evidence has suggested a possible intriguing function for the entire “early protein group” of HPV16 in inhibiting the autophagy onset and consequently in determining post-infection virus survival, the single contribution of each of the early proteins and the possible molecular mechanisms involved remain to be clarified.

The early HPV16 protein E5 (16E5) cooperates with the viral oncogenes 16E6 and 16E7 during HPV16-associated cervical carcinogenesis [6, 10, 11]. 16E5 is a multifunctional protein whose oncogenic activity is related to its ability to interfere with the expression and signaling of several receptor tyrosine kinases (RTKs), prevalently by deregulating their sorting to the endocytic degradative pathway through different mechanisms [11].

Among the HPV16 early proteins, 16E5 might represent the best candidate as deregulator of the autophagic process, since this viral protein is able to down-regulate the expression of the keratinocyte growth factor receptor (KGFR/FGFR2b) [12, 13], the exclusive epithelial splicing variant of the fibroblast growth factor receptor 2 (FGFR2) [14, 15] and we have very recently reported that this receptor induces autophagy in keratinocytes [16]. In addition, we have also proposed that 16E5 interferes with the keratinocyte differentiation induced by KGF/KGFR signaling [13] and that the receptor inductive effect on autophagy is required for its ability to trigger early differentiation [16]. Therefore, based on these evidences, it is reasonable to assume that 16E5 might interfere with autophagy through KGFR. With the initial aim to investigate the possible effects of 16E5 on the KGF-induced autophagy in human keratinocytes, we not only confirmed our starting hypothesis, but also we found that the viral protein has a more general impact on autophagy, which involves the impairment of both p53-mediated and p53-independent transcriptional regulation of the process.

## RESULTS

### HPV16 E5 inhibits both KGF-triggered and KGF-independent autophagy in human keratinocytes

**Figure 1:**
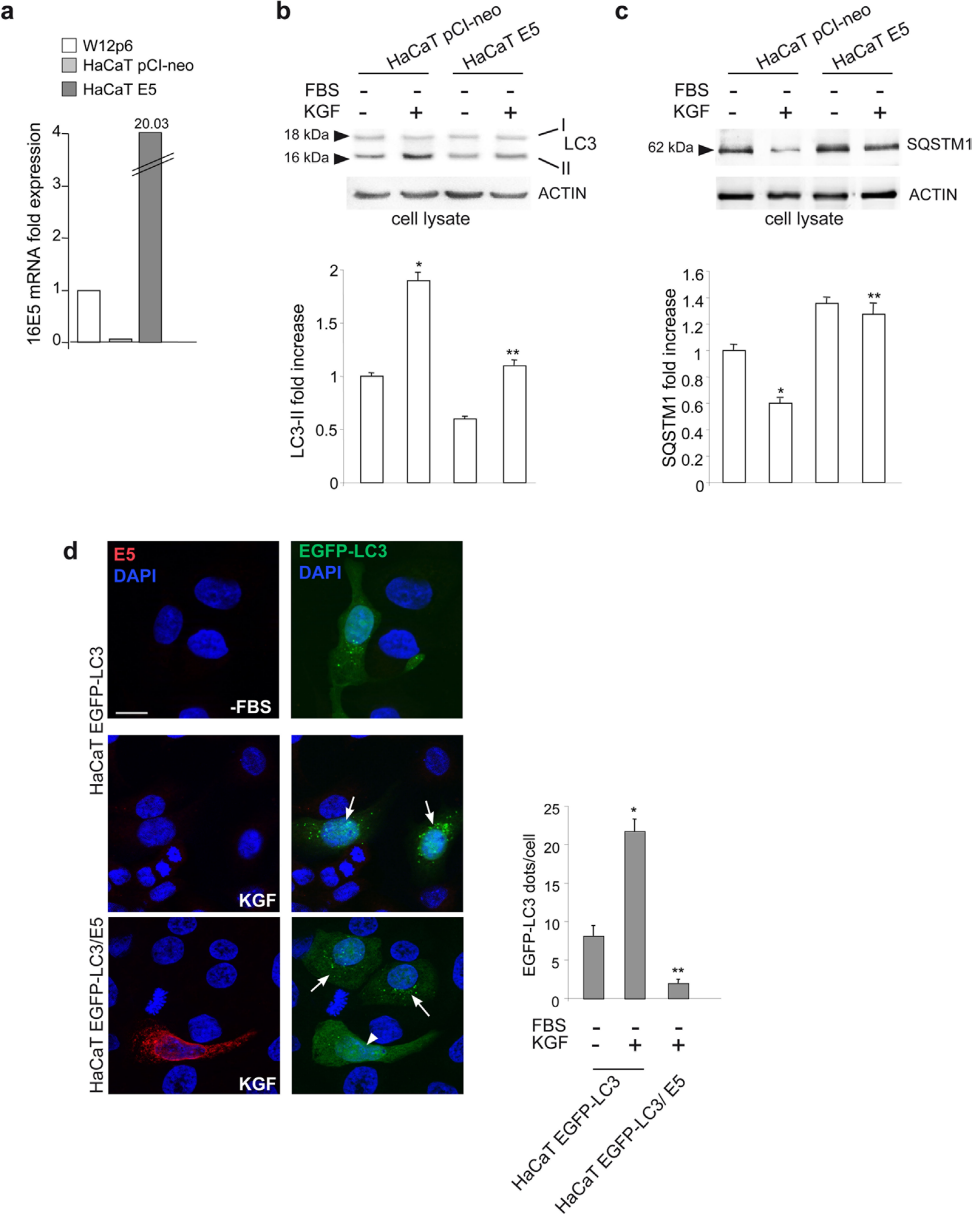
16E5 inhibits KGF-induced autophagy **(a)** HaCaT cells were transiently transfected with pCI-neo E5-HA expression vector (HaCaT E5) or with the empty vector alone (HaCaT pCI-neo). The 16E5 mRNA transcripts, quantitated by real-time relative RT-PCR and normalized with respect to those detected in the HPV16-positive cervical epithelial cell line W12 at the passage 6 (W12p6), are highly expressed only in HaCaT E5 cells. **(b, c)** HaCaT E5 and HaCaT pCI-neo cells were serum-starved in the presence or absence of KGF 100 ng/ml for 24 h. Western blot analysis shows that, upon KGF stimulation, the LC3-II band is reduced (b), while the SQSTM1 band is enhanced (c), in HaCaT E5 cells compared to HaCaT pCI-neo cells. The equal loading was assessed using anti-β actin antibody. For the densitometric analysis, the values from 3 independent experiments were normalized, expressed as fold increase and reported in graph as mean values ± standard deviation (SD). Student *t* test was performed and significance levels have been defined as *p* < 0.05: (b, c) *p < 0.05 vs the corrisponding unstimulated cells, **p < 0.05 vs the corresponding HaCaT pCI-neo cells. **(d)** HaCaT cells were transiently cotransfected with pEGFP-C2-LC3 construct and pCI-neo E5-HA (HaCaT EGFP-LC3/E5) or pCI-neo empty vector (HaCaT EGFP-LC3) before stimulation with KGF as above. Immunofluorescence was performed using anti-HA monoclonal antibody (red), to visualize 16E5, and cell nuclei were stained with DAPI. Upon KGF treatment, the number of LC3-positive dots per cell is increased in HaCaT EGFP-LC3 cells and in HaCaT EGFP-LC3/E5 cells not showing 16E5 staining (arrows), but is reduced in HaCaT EGFP-LC3/E5 cells strongly labeled for 16E5 (arrowhead) if compared to serum-starved HaCaT EGFP-LC3 cells. The quantitative analysis was performed as described in the materials and methods and results are expressed as mean values ± standard errors (SE). Student *t* test was performed and significance levels have been defined as *p* < 0.05: *p < 0.001 vs the corresponding serum starved-cells, **p < 0.001 vs the corresponding HaCaT EGFP-LC3 cells. Bar: 10 μm

Since we have recently demonstrated that 16E5 down-regulates KGFR [12, 13], whose ligand-specific activation triggers autophagy in keratinocytes [16], here we analyzed the effects of 16E5 ectopic expression on KGF-triggered autophagy in the human keratinocyte HaCaT cell line, spontaneously immortalized from a primary culture of keratinocytes [17]. To this aim, cells were transiently transfected with pCI-neo E5-HA expression vector [18] (HaCaT E5) or with the empty vector alone (HaCaT pCI-neo). The expected high expression of 16E5 mRNA transcript levels in HaCaT E5 [13] was first confirmed by real-time relative RT-PCR and normalized with respect to the levels of the viral protein transcript in the HPV16-positive cervical epithelial cell line W12 [19] at the passage 6 (W12p6) (Figure 1a). Then, to investigate the possible effects of 16E5 expression on KGF-induced autophagy, HaCaT pCI-neo and HaCaT E5 cells were serum-starved in the presence or absence of KGF for 24 h. Both the growth factor concentration and the single time point of treatment have been previously selected as optimal experimental conditions for an efficient autophagic induction in HaCaT cells [16]. The amount of the well-established autophagosome marker membrane-associated microtubule associated protein 1 light chain 3-II (LC3-II) was monitored by western blot analysis. The results showed that, after KGF stimulation, the increase of the 16 kDa band corresponding to LC3-II marker, evident in HaCaT pCI-neo cells (Figure 1b), appeared significantly reduced in HaCaT E5 cells (Figure 1b), indicating that the KGF-induced autophagosome formation was counteracted by the presence of 16E5.

To more carefully investigate the effect of 16E5 expression on the autophagic flux, the levels of the well-known autophagy substrate SQSTM1/p62 (sequestosome 1) were estimated by western blot analysis. The evident decrease of the 62 kDa band corresponding to SQSTM1, observed in HaCaT pCI-neo cells upon KGF stimulation, appeared significantly recovered in HaCaT E5 cells (Figure 1c), indicating that the SQSTM1 degradation was prevented in 16E5-expressing cells. Moreover, the accumulation of this autophagic substrate seems to indicate that the viral protein acts by inhibiting the formation of new autophagosomes, rather than by accelerating their turnover.

The interference of 16E5 expression on the enhanced autophagy was also investigated by the widely accepted fluorescence approach. To directly quantify the autophagosome number in cells ectopically expressing 16E5 and to easily compare it with cells which did not express the viral protein, HaCaT cells were transiently cotransfected with pEGFP-C2-LC3 construct and pCI-neo E5-HA (HaCaT EGFP-LC3/E5) or pCI-neo empty vector (HaCaT EGFP-LC3) as a control. Cells were then treated with KGF as above, fixed, permeabilized and nuclei were stained with DAPI. Quantitative immunofluorescence analysis was performed using anti-HA monoclonal antibody to visualize the viral protein. Results clearly showed that, upon KGF treatment, a significant increase of the LC3-positive dots per cell, corresponding to the assembled autophagosomes, was evident in HaCaT EGFP-LC3 cells (Figure 1d, middle panels, arrows) or in HaCaT EGFP-LC3/E5 cells not showing 16E5 expression (Figure 1d, lower panels, arrows), while this increase appeared significantly abolished in HaCaT EGFP-LC3/E5 cells highly expressing 16E5 (Figure 1d, lower panels, arrowhead). Interestingly, in these latter cells, the number of LC3 positive dots was even lower than that observed in serum-starved control cells (Figure 1d, upper panels). Since serum starvation is *per se* an autophagic stimulus, these results suggest that 16E5 might play a more general role, independent on KGF, in autophagy impairment.

**Figure 2:**
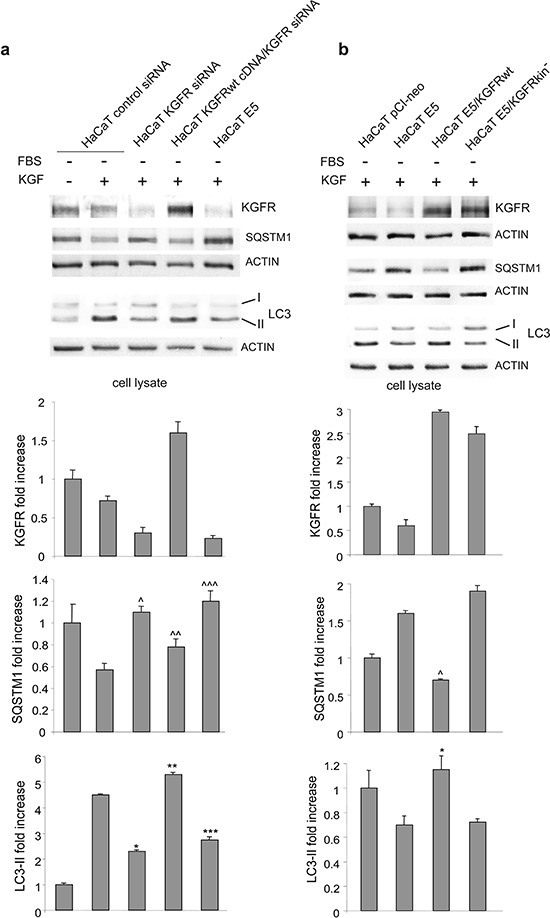
The inhibitory effect of 16E5 on KGF-triggered autophagy depends on KGFR expression and signaling **(a)** HaCaT cells were transfected with 16E5 cDNA (HaCaT E5), with a small interfering RNA for FGFR2/Bek (HaCaT KGFR siRNA) or with an unrelated siRNA (HaCaT control siRNA) as control. Alternatively cells were cotransfected with KGFRwt cDNA and with KGFR si RNA. Cells were then stimulated with KGF as above. Western blot analysis shows that, upon KGF stimulation, both KGFR and LC3-II bands are reduced, while the SQSTM1 band is increased either in 16E5-transfected and KGFR-depleted cells. **(b)** Cells were transiently transfected with 16E5 (HaCaT E5) or cotransfected with 16E5 and pCI-neo vector containing human KGFRwt (HaCaT E5/KGFRwt) or the kinase negative mutant KGFRY656F/Y657F (HaCaT E5/KGFRkin^−^) and stimulated with KGF as above. Western blot analysis shows that the decrease of LC3-II as well as the increase of SQSTM1 induced by 16E5 expression is counteracted only by KGFRwt overexpression. The densitometric analysis and Student *t* test were performed as reported above: (a) ^, ^^^, ***p < 0.05 and *p < 0.01 vs the corrisponding HaCaT control siRNA cells, ^^*p* < 0.05 and **p < 0.01 vs the corrisponding HaCaT KGFR siRNA cells; (b) *, ^*p* < 0.05 vs HaCaT E5 cells.

To clarify whether the inhibition of KGF-dependent autophagy induced by 16E5 is directly related to its previously reported ability to down-regulate KGFR expression and signaling [12, 13], we first compared the effects of 16E5 expression to those induced by KGFR depletion. HaCaT cells were singly transfected with 16E5 cDNA or with a small interfering RNA for FGFR2/Bek (HaCaT KGFR siRNA) or an unrelated siRNA (HaCaT control siRNA) as control and then stimulated with KGF as above. In addition, in order to assess whether the possible effects induced by KGFR depletion can be counteracted by its simultaneous forced expression, cells were also doubly transfected with KGFR siRNA and pCI-neo vector containing human KGFRwt (HaCaT KGFRwt cDNA/KGFR siRNA). Western blot analysis showed that both 16E5-transfected and KGFR-depleted cells not only displayed receptor down-regulation as expected [13], but also a significant decrease of LC3-II levels as well as a block of SQSTM1 degradation in response to KGF (Figure 2a). Moreover, the inhibitory effects on autophagy induced by KGFR depletion was reverted by the simultaneous overexpression of the receptor (Figure 2a). Thus, 16E5 expression and KGFR silencing appeared to affect the autophagic process in a similar manner. To further demonstrate the receptor involvement on the 16E5 effect on autophagy, we performed KGFR forced overexpression in the presence of the viral protein: to this aim, cells were transiently cotransfected with 16E5 (HaCaT E5) and KGFRwt (HaCaT E5/KGFRwt) or the kinase negative mutant KGFRY656F/Y657F (HaCaT E5/KGFRkin^−^). After transfection, cells were stimulated with KGF as above. Western blot analysis clearly showed that the 16E5-induced decrease of LC3-II levels as well as SQSTM1 accumulation was reverted by the expression of KGFRwt, but not by that of KGFRkin- (Figure 2b). Therefore, KGFR forced expression and receptor activation are sufficient to counteract the inhibitory effect of 16E5 on the autophagy upon growth factor treatment. These results demonstrate that, although the molecular mechanisms remain to be clarified, 16E5 appears to impact the pro-autophagic KGFR pathway through the down-regulation of the receptor.

**Figure 3:**
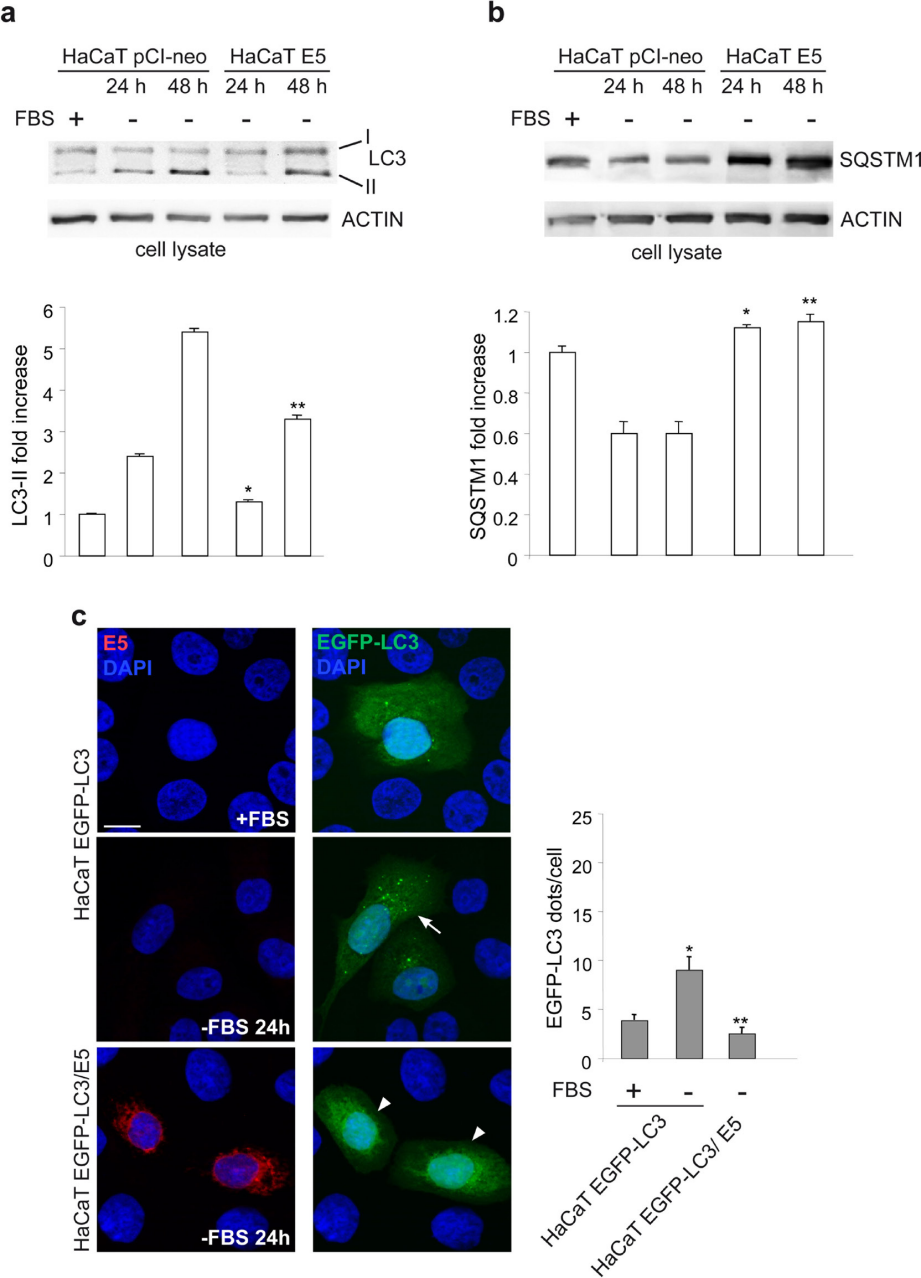
16E5 inhibits also the serum starvation-induced autophagy **(a, b)** HaCaT pCI-neo and HaCaT E5 cells were kept in complete medium or serum-starved for 24 h or 48 h. Western blot analysis shows that in HaCaT E5 cells the serum deprivation-induced progressive increase of LC3-II band is reduced, while the decrease of SQSTM1 is blocked. The densitometric analysis and Student *t* test were performed as above: (a) *p < 0.01 vs the corresponding HaCaT pCI-neo cells, **p < 0.05 vs the corresponding HaCaT pCI-neo cells; (b) *, **p < 0.05 vs the corresponding HaCaT pCI-neo cells. **(c)** Immunofluorescence analysis performed in HaCaT EGFP-LC3 and HaCaT EGFP-LC3/E5 cells serum-starved as above shows no increase in LC3-positive dots in cells expressing 16E5 (arrowheads) compared to HaCaT EGFP-LC3 (arrow). The quantitative analysis and Student *t* test were performed as above: *p < 0.005 vs the corresponding serum cultured-cells, **p < 0.005 vs the corresponding HaCaT EGFP-LC3 cells. Bar: 10 μm

To deeper investigate the possibility that 16E5 might play a more general role in autophagy impairment, the possible effects of its ectopic expression were analysed in cells subjected to serum starvation, an autophagic stimulus in which the contribution of KGFR signaling is completely excluded. HaCaT pCI-neo and HaCaT E5 cells were kept in complete medium or serum-starved for the two time points (24 h and 48 h) previously selected as optimal conditions for an efficient induction of autophagy in HaCaT cells [16]. Western blot analysis performed as above showed that in HaCaT E5 cells the progressive increase of LC3-II marker was significantly affected (Figure 3a), while the SQSTM1 degradation was totally abolished (Figure 3b). The interference of 16E5 expression was also investigated by immunofluorescence as above. The results showed that the significant increase of the LC3-positive dots induced by 24 h of serum starvation, evident in HaCaT EGFP-LC3 (Figure 3c, arrow), was completely blocked in HaCaT EGFP-LC3/E5 (Figure 3c, arrowheads), unequivocally demonstrating that the presence of the viral protein prevents the increase of autophagosomes in response to serum deprivation. Thus, independently from the stimulus that triggers the process, 16E5 appears to generally interfere with autophagy.

**Figure 4:**
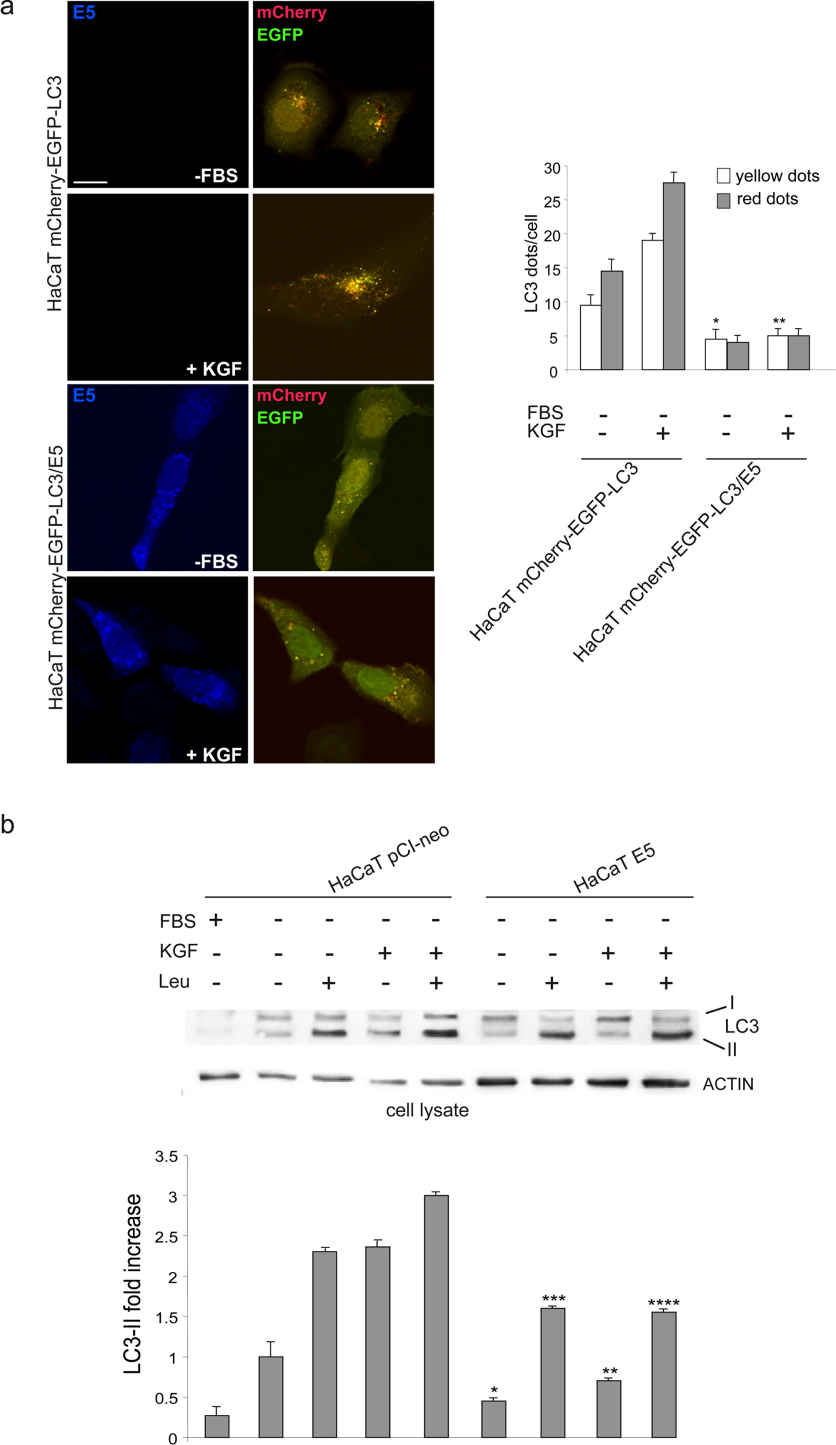
16E5 inhibits autophagosome assembly **(a)** HaCaT mCherry-EGFP-LC3 and HaCaT mCherry-EGFP-LC3/E5 cells were serum-starved or treated with KGF as above. Immunofluorescence analysis shows that in E5 expressing cells the number of yellow dots corresponding to newly assembled autophagosomes is decreased, while the red dots corresponding to autolysosomes are not increased compared to control cells. The quantitative analysis and Student *t* test were performed as above: *p < 0.05, **p < 0.01 vs the corresponding HaCaT mCherry-EGFP-LC3 cells. Bar: 10 μm **(b)** HaCaT pCIneo and HaCaT pCI-neo/E5 cells were serum-starved or treated with KGF in the presence or absence of leupeptin (LEU) as reported in materials and methods. Western blot shows that in 16E5 expressing cells the LC3-II levels are significantly reduced also in the presence of the inhibitor of the lysosomal degradation. The densitometric analysis and Student *t* test were performed as reported above: * and **p < 0.05 vs the corresponding HaCaT pCI-neo cells, *** and ****p < 0.01 vs the corresponding HaCaT pCI-neo cells.

In order to confirm that 16E5 is able to impact the autophagy on-rate, rather than the autophagy off-rate, as already indicated above by SQSTM1 monitoring, immunofluorescence experiments were performed doubly transfecting HaCaT cells with 16E5 and a pDest-mCherry-EGFP-LC3 tandem construct [20]. In fact, mCherry-EGFP-LC3 is an autophagic flux sensor, since EGFP fluorescence is quenched in acidic environments, whereas mCherry is an acidic-stable fluorescent tag: the nascent autophagosomes are both red and green (yellow) labeled, whereas the acidic autolysosomes appear red, as a consequence of the EGFP quenching. Quantitative immunofluorescence analysis performed upon either serum deprivation and KGF stimulation showed that 16E5 expression led to a significant decrease in the number of yellow dots per cells corresponding to newly assembled autophagosomes (Figure 4a), while the quantity of red dots corresponding to autophagosomes flowed in the lysosomes was not affected (Figure 4a). The inhibitory effect of 16E5 on autophagosome formation was further confirmed monitoring the LC3-II levels in presence or absence of the well known lysosomal protease inhibitor leupeptin (LEU, Figure 4b), which inhibits the vacuolar type H^+^-ATPase (v-ATPase) complex necessary for lysosomal acidification [21]. Western blot analysis performed upon serum deprivation or KGF stimulation showed that 16E5 expression significantly decreases LC3-II levels also in the presence of the inhibitor of the autophagic flux (Figure 4b), confirming that, independently from the stimulus which triggers autophagy, 16E5 exerts an inhibitory role in the autophagosome assembly.

**Figure 5:**
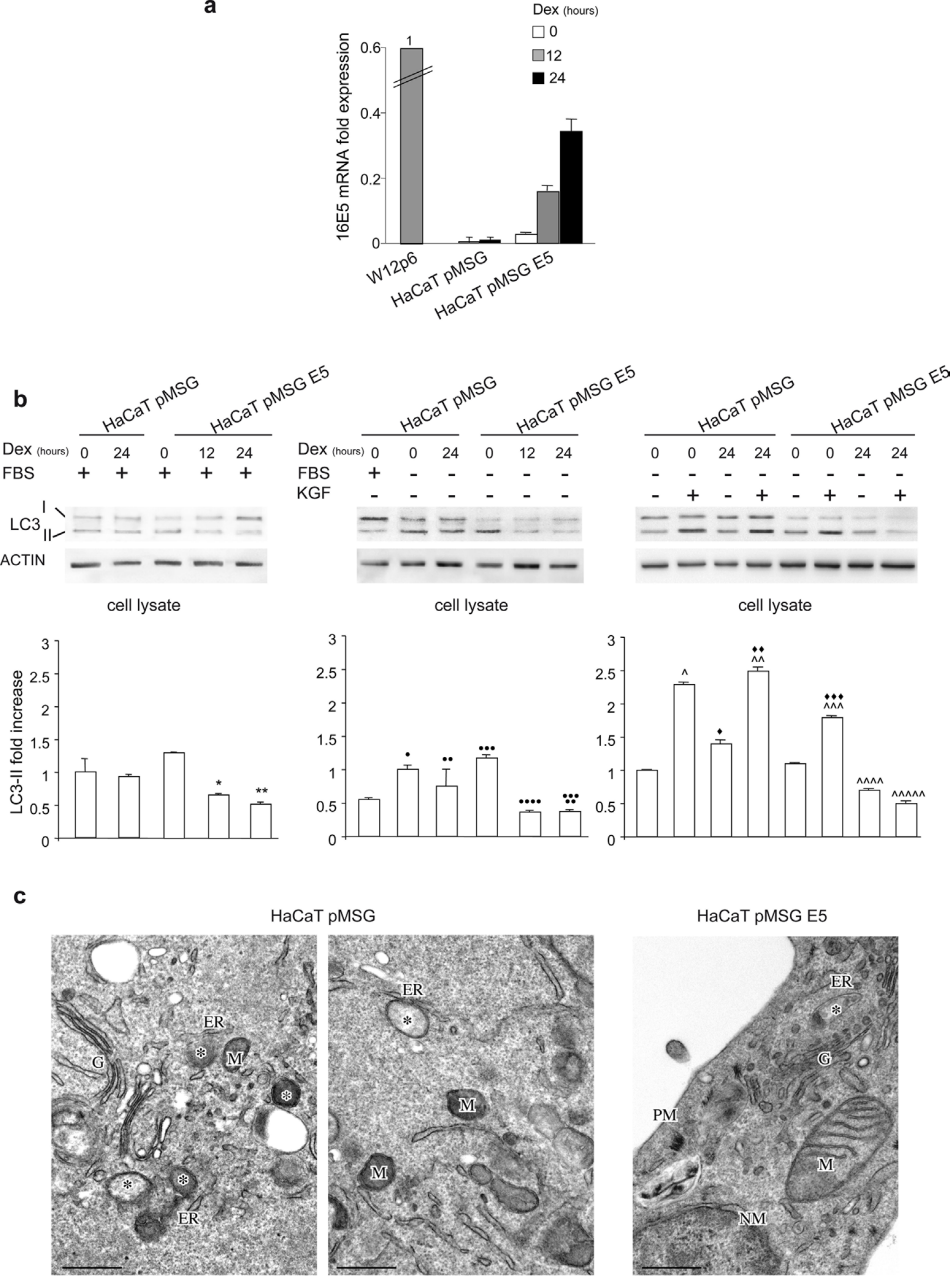
Impairment of autophagy in cells stably expressing 16E5 **(a)** HaCaT pMSG and HaCaT pMSG E5 cells were left untreated (0 h) or treated with Dex for 12 h or 24 h. The 16E5 the increasing mRNA transcript levels were quantitated by real-time relative RT-PCR and normalized with respect to those detected in W12p6 cells. **(b)** Cells were kept in complete medium or either serum-starved or stimulated with KGF for 24 h in presence or absence of Dex induction. Western blot analysis shows that in serum-kept cells (left panel) the very weak band corresponding to LC3-II is decreased at 12 h and 24 h of Dex treatment in HaCaT pMSG E5 cells, while no changes in the band intensity are observed in HaCaT pMSG cells. Upon serum starvation (middle panel) or KGF stimulation (right panel) the evident increase of LC3-II band is abolished by Dex treatment only in HaCaT pMSG E5 cells, but not in control cells. In absence of Dex treatment, the increase of LC3-II protein induced KGF is lower in HaCaT pMSG E5 than in control cells. The densitometric analysis and Student *t* test were performed as above: *, **p < 0.05 vs the corresponding Dex-untreated cells, • *p* < 0.05 vs the corresponding serum-cultured cells, •• NS vs the corresponding Dex-untreated cells, ••• NS vs the corresponding HaCaT pMSG cells, ••••, ••••• *p* < 0.05 vs the corresponding Dex-untreated cells, ^, ^^*p* < 0.05 vs the corresponding KGF-unstimulated cells, ♦♦♦NS vs the corresponding Dex-untreated cells, ^^^*p* < 0.01 vs the corresponding KGF-unstimulated cells, ^^^^*p* < 0.05 vs the corresponding Dex-untreated cells, ^^^^^*p* < 0.01 vs the corresponding Dex-untreated cells, ♦♦♦*p* < 0.05 vs the corresponding HaCaT pMSG cells. **(c)** Ultrastructural analysis of HaCaT pMSG and HaCaT pMSG E5 cells stimulated with KGF for 24 h in presence of Dex: the number of double-membrane autophagic vacuoles (asterisks) is lower in HaCaT pMSG E5 (right panel) compared to HaCaT pMSG cells (left and middle panels). ER, endoplasmic reticulum; M, mitochondrion; NM, nuclear membrane; PM, plasma membrane; G, Golgi complex. Bars: 0.5 μm.

In order to define whether the effect of 16E5 on autophagy could be dose-dependent, we took advantage of the use of HaCaT cells stably transfected with the construct pMSG 16E5 (HaCaT pMSG E5) [22], in which the expression of the viral protein can be progressively induced, in a time-dependent manner, by treatment with dexamethasone (Dex). The HaCaT pMSG cells were used as negative control. Cells were left untreated (0 h) or treated with Dex for 12 h or 24 h, and the increasing 16E5 mRNA transcript levels were quantitated by real-time relative RT-PCR. The mRNA amounts were normalized respect to the levels expressed in W12p6 cells. The results clearly indicated that in HaCaT pMSG E5 cells, which expressed very low levels of 16E5 mRNA also in absence of Dex treatment [22–24], the increasing levels of 16E5 mRNA after Dex stimulation remain lower than those observed in the endogenous model of W12p6 cells (Figure 5a). To first analyse the impact of the progressive expression of 16E5 on basal autophagy, cells were kept in complete medium and treated with Dex as above. Western blot analysis showed that in HaCaT pMSG E5 cells the low expression of LC3-II protein was decreased already after 12 h of Dex treatment and no further decrease was observed after 24 h (Figure 5b, left panel). Interestingly, no changes on LC3-II amounts were induced by Dex in control cells (Figure 5b, left panel), demonstrating that the inhibitory effect observed in HaCaT pMSG E5 cells can be specifically ascribed to 16E5 expression. In addition, these results indicate that the observed inhibition of autophagy does not occur only when the viral protein is overexpressed.

Then, our attention was shifted from the basal to induced-autophagy. Western blot analysis showed that the evident increase of LC3-II levels induced by both serum starvation (Figure 5b, middle panel) and KGF stimulation (Figure 5b, right panel) appeared completely abolished upon Dex treatment in HaCaT pMSG E5 cells; again, no effects were found in control cells, confirming the exclusive role of 16E5. Interestingly, in absence of Dex treatment, the increase of LC3-II protein caused by KGF appeared significantly lower in HaCaT pMSG E5 than in control cells (Figure 5b, right panel), implying that the low levels of 16E5 expressed by these cells in Dex-untreated conditions (see Figure 5a) were sufficient to interfere with the enhancement of autophagy induced by KGF (Figure 5b, right panel). The ability of 16E5 to inhibit autophagy was also analyzed in detail by transmission electron microscopy (TEM). The ultrastructural observations revealed that the double-membrane autophagic vacuoles (Figure 5c, asterisks), varying in shapes and frequently tightly apposed to endoplasmic reticulum cisternae, were numerous in HaCaT pMSG cells treated with KGF in the presence of Dex (Figure 5c, left and middle panels) and drastically reduced in HaCaT pMSG E5 subjected to the same treatment (Figure 5c, right panel). Thus, the ultrastructural analysis unequivocally demonstrated that the 16E5-induced impairment of autophagy shown by biochemical or immunofluorescence approaches corresponds to a real reduction in the number of double-membrane vacuolar structures morphologically identifiable as autophagosomes.

**Figure 6:**
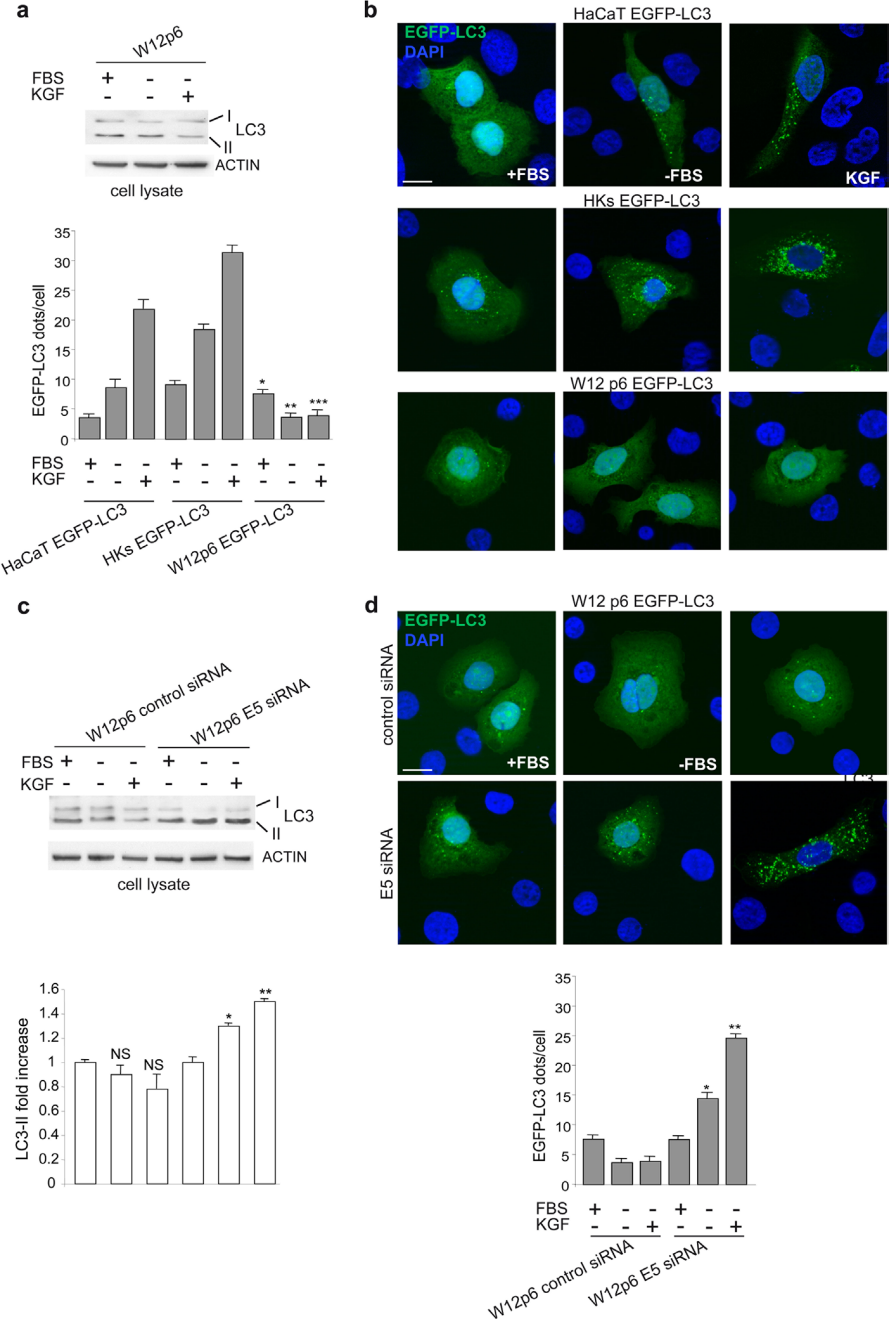
The unresponsiveness of W12p6 cells to autophagic stimuli depends on 16E5 expresssion **(a)** Cells were kept in complete medium or serum-starved in the presence or absence of KGF for 24 h. Western blot show no changes in the levels of LC3-II marker in W12p6 cells upon both serum deprivation or KGF stimulation. **(b)** W12p6 cells were transfected with EGFP-LC3 and treated as above. Fluorescence analysis show an increase in the number of LC3-positive dots per cell after serum starvation and/or KGF stimulation in HaCaT EGFP-LC3 and HKs EGFP-LC3 control cells, but not in W12p6 EGFP-LC3 cells. The quantitative analysis and Student *t* test were performed as above: *p < 0.05 vs the corresponding EGFP-LC3 HaCaT cells; **, ***p > 0.001 vs the corresponding EGFP-LC3 HaCaT cells or vs the corresponding EGFP-LC3 HKs. **(c)** W12p6 cells were transfected with E5 siRNA or with an unrelated siRNA as control and treated as above. The LC3-II levels are progressively increased by serum deprivation and by KGF in W12p6 E5 siRNA, while no changes are observed in W12p6 control siRNA The densitometric analysis and Student *t* test were performed as above: NS vs the corresponding serum-cultured cells; *, **p < 0.05 vs the corresponding W12p6 control siRNA cells. **(d)** W12p6 cells were cotransfected with EGFP-LC3 and with E5 siRNA or with a control siRNA and treated as above. Fluorescence approaches show a significant increase of LC3-positive dots in 16E5-depleted W12 cells upon serum deprivation and even more upon KGF stimulation. No increase is found in control siRNA-transfected cells. *, **p < 0.001 vs the corresponding control siRNA. Bars 10 μm

To verify whether the viral protein exerts the inhibitory effect on autophagy also in the presence of the HPV16 full-length genome, as it occurs in the context of cervical carcinogenesis, we used the well established *in vitro* model of cervical W12p6 cells, containing episomal HPV16. Western blot analysis clearly showed that no detectable changes in LC3-II marker levels could be found in these cells upon starvation or KGF treatment (Figure 6a). Fluorescence approaches were also performed using W12p6 cells transiently transfected with pEGFP-C2-LC3 (W12p6 EGFP-LC3). HaCaT cells or primary cultures of normal human keratinocytes (HKs) transiently transfected with EGFP-LC3 (HaCaT EGFP-LC3 and HKs EGFP-LC3) were used as controls. The results clearly demonstrated that, differently from control cells (Figure 6b), W12p6 EGFP-LC3 cells did not show any increase in the number of LC3-positive dots per cell after serum starvation and/or KGF stimulation (Figure 6b).

In order to investigate whether the lack of responsiveness to the autophagic stimuli detected in the endogenous context of W12p6 cells may be due to 16E5 expression, the effect of specific depletion of the viral protein was analyzed by siRNA transfection. We first confirmed the efficient depletion of the 16E5 protein in E5 siRNA-transfected cells performing experiments on HaCaT cells cotransfected with E5-HA cDNA and E5 siRNA in which the efficiency of 16E5 silencing was verified through western blot analysis using anti-HA monoclonal antibody (Supplementary Figure 1). Then, W12p6 cells were transfected with the specific 16E5 siRNA or with an unrelated siRNA as control and the autophagic process was stimulated by serum starvation or KGF treatment as above. Western blot analysis clearly showed that both the autophagic stimuli significantly increased the LC3-II levels only in 16E5-depleted cells (Figure 6c). Consistent with the biochemical results, fluorescence approaches revealed that a significant increase of LC3-positive dots was evident in 16E5-depleted W12 cells upon serum deprivation and KGF stimulation (Figure 6d), while no increase was found in control siRNA-transfected cells (Figure 6d). These results strongly indicated that in W12p6 cells, which are the most representative model of cervical cancerogenesis, the observed unresponsiveness to autophagic stimuli can be specifically ascribed to 16E5 expression.

### 16E5 interferes with the transcriptional regulation of autophagy through the impairment of p53 function

**Figure 7:**
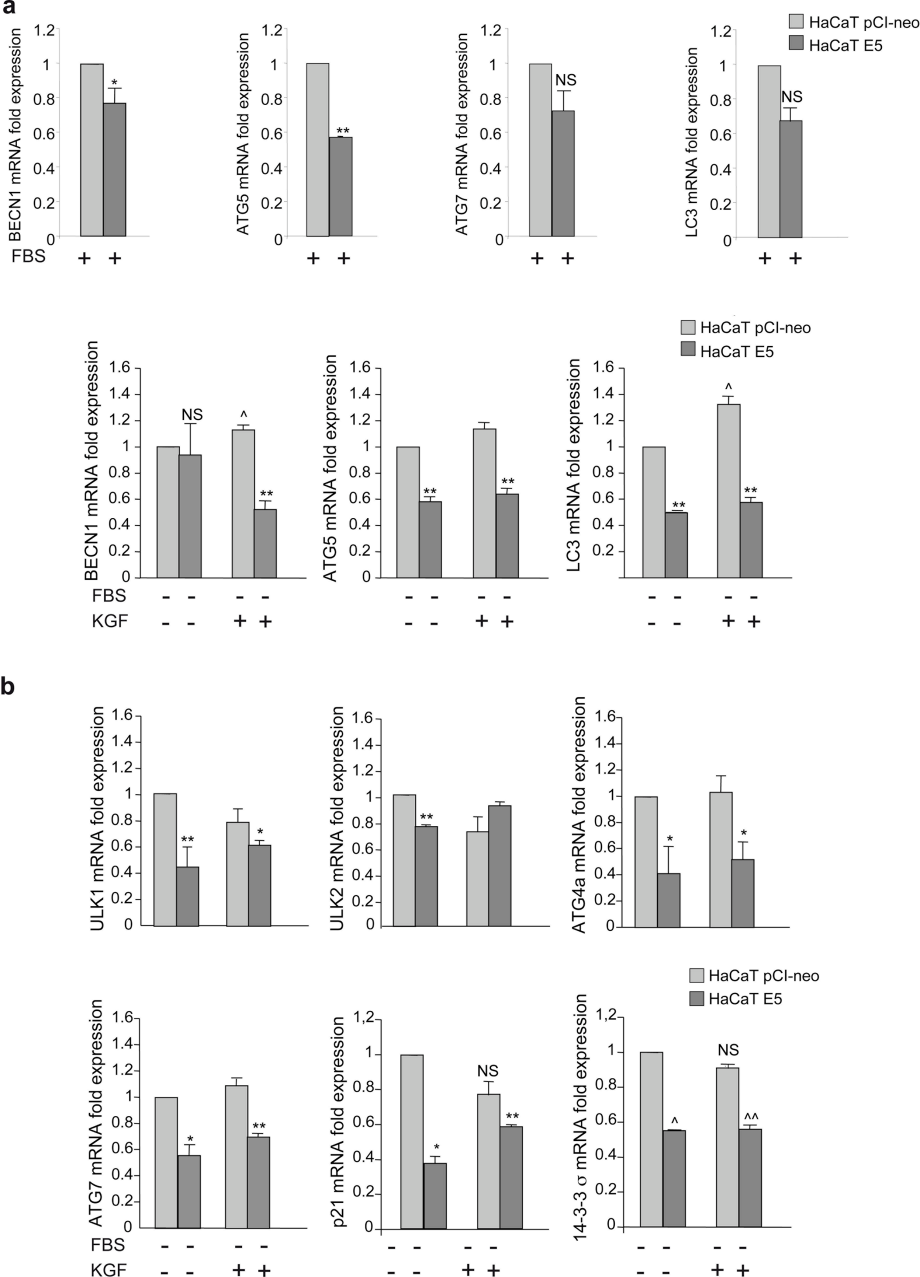
16E5 expression down-modulates the autophagy gene expression in HaCaT cells **(a, b)** HaCaT pCI-neo and HaCaT E5 cells were kept in complete medium or serum-starved or stimulated with KGF as above. Real-time relative RT-PCR of key regulatory autophagy genes (a) or p53-target autophagic (ULK1, ULK2, ATG4a, ATG7) or autophagy-independent (p21, 14–3-3-σ) genes (b). Results are expressed as mean ± standard error (SE) from three different experiments in triplicate. Student *t* test was performed and significance levels have been defined as *p* < 0.05: (a) *p < 0.05 and **p < 0.01 vs the corresponding HaCaT pCI-neo cells, NS vs the corresponding HaCaT pCI-neo cells, ^*p* < 0.05 vs the corresponding KGF-unstimulated cells. (b) *, **, ^, ^^*p* < 0.05 vs the corresponding HaCaT pCI-neo cells, NS vs the corresponding KGF-unstimulated cells.

Since it has been demonstrated that 16E5 is able to affect the expression of several host genes [25, 26] and growing evidences indicate that autophagy is not only post-translationally regulated, but also transcriptionally controlled [27–29], here we investigated whether 16E5 might interfere with autophagy by affecting the autophagic gene expression. To this aim, the mRNA transcript levels of different crucial autophagic genes acting at different steps of the process (BECN1, ATG5 and LC3) were estimated by real-time relative RT-PCR in HaCaT E5 cells and normalized respect to the levels detected in HaCaT pCI-neo cells. In cells kept in complete medium, BECN1 and ATG5, but not LC3 or ATG7, appeared down-regulated by 16E5 expression (Figure 7a, upper panels). Moreover, when autophagy is stimulated by serum starvation or KGF treatment, a drastic significant decreased expression of all genes examined, except BECN1 in serum-deprived cells, was evident (Figure 7a, lower panels). Thus, 16E5 down-regulates autophagy gene expression when the process is induced as well as under basal conditions. Interestingly, in agreement with our previous biochemical observations [16], KGF stimulation slightly but significantly increased the expression of BECN1 and LC3, while that of ATG5 seemed unaffected (Figure 7a, lower panels) indicating that KGF/KGFR signaling plays a role in the transcriptional control of autophagy.

The p53 protein has been recently identified as a possible transcriptional inductor of the autophagic program [29] and several autophagy genes are found to be positively regulated by p53 also in HaCaT cells [30], although these cells are known to express mutant p53 alleles [30]. Therefore, in order to assess whether 16E5 might negatively affect the transcriptional program of autophagy also interfering with the expression of a set of p53-regulated autophagy core machinery genes (ULK1, ULK2, ATG4a, ATG7) [29], we analyzed their transcript levels as above. Real-time relative RT-PCR showed that all the p53-regulated genes, with the only exclusion of ULK2, were significantly down-regulated by 16E5 expression upon either serum starvation or KGF stimulation (Figure 7b). Thus, during induced-autophagy, the viral protein is able to repress the expression of several autophagic genes, some of which are specific targets of p53. In addition, since no changes in the mRNA levels of the examined p53-target genes were observed upon KGF treatment (Figure 7b), these results show that the KGFR transcriptional regulation of autophagy is p53-independent.

In order to verify if 16E5 could interfere with the transcriptional regulation of autophagy inducing impairment of p53 function, the expression of two well established p53 downstream target genes, such as p21 and 14–3-3σ, was analyzed in HaCaT E5 and HaCaT pCI-neo cells upon serum starvation or KGF stimulation. RT-PCR analysis showed in 16E5-expressing cells a significant decrease of p53 target gene expression (Figure 7b, lower panels), suggesting that 16E5 could be able to transcriptionally impair autophagy also interfering with p53 function. In contrast, consistent with the results described above, the stimulation with KGF was able to induce no significant changes of p21 or 14–3-3σ expression (Figure 7b, lower panels), further confirming that the induction of autophagy by KGF does not involve the p53 regulation.

**Figure 8:**
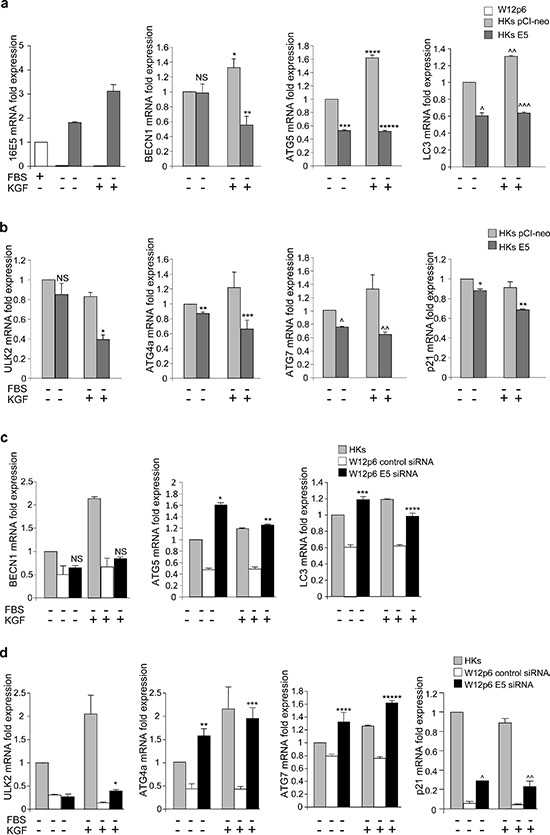
16E5 depletion in the W12p6 cervical carcinogenesis model restores the autophagic gene expression **(a, b)** HKs pCI-neo and HKs E5 cells were kept in complete medium or serum-starved or stimulated with KGF as above. Real-time relative RT-PCR of key regulatory autophagy genes (a) or p53-target genes (b). **(c, d)** W12p6 control siRNA and W12p6 E5 siRNA cells and HKs were treated as above. Real-time relative RT-PCR of key regulatory autophagy genes (c) or p53-target genes (d). Results are expressed as mean ± standard error (SE). Student *t* test was performed and significance levels have been defined as above: (a) *p < 0.05 and **** and ^^*p* < 0.005 vs the corresponding KGF-unstimulated cells, **p < 0.05, ^*p* < 0.005 and ***, *****, ^^^*p* < 0.001 vs the corresponding HKs pCI-neo cells, NS vs the corresponding HKs pCI-neo cells; (b) *, **, ***, ^^*p* < 0.05 and ^*p* < 0.001 vs the corresponding HKs pCI-neo cells, NS vs the corresponding HKs pCI-neo cells; (c) *, **p < 0.001 and ***, ****p < 0.05 vs the corresponding W12p6 control siRNA, NS vs the corresponding W12p6 control siRNA; (d) *, **, ***, ^^*p* < 0.05 and ****, *****, ^*p* < 0.005 vs the corresponding W12p6 control siRNA.

In order to verify if the ability of 16E5 to transcriptionally regulate autophagy is a general phenomenon, we examined the expression of the autophagic genes in primary human keratinocytes transiently transfected with 16E5 (HKs E5) or with the pCI-neo empty vector (HKs pCI-neo) as control. The results showed that, also in primary cultures, the expression of 16E5 appeared to down-regulate most of the p53-independent (Figure 8a) and p53-regulated (Figure 8b) autophagy genes, as well as that of the main p53-target gene p21 (Figure 8b). Consistently with the results obtained in HaCaT cells, also in HKs E5 cells the stimulation with KGF significantly increased the expression of BECN1, ATG5 and LC3, while p53-target genes appeared unaffected (Figure 8a, 8b), confirming that KGF appears to exert a transcriptional control only on the p53-independent autophagy genes.

To assess whether the repression of the autophagic gene transcription induced by 16E5 could be observed in the presence of HPV16 full-length genome and to analyze whether this effect could be directly due to the E5 viral protein expression, all the previously examined genes were re-analyzed in W12p6 cells transfected with a specific E5 siRNA or with un unrelated siRNA. The mRNA levels of the different genes in HKs were used as normalizers. Real-time relative RT-PCR showed that the very low levels of most of the genes in W12p6 control siRNA cells (Figure 8c, 8d) were recovered upon 16E5 depletion (Figure 8c, 8d). These results strongly suggested that the decreased expression of both p53-regulated and p53-independent autophagic genes observed in W12 cells compared to HKs can be directly ascribed to 16E5 expression.

## DISCUSSION

Impairment of the host cell autophagic response is a general strategy used by viruses during the early steps of infection in order to ensure their intracellular survival and subsequent replication [5]. In the case of human papillomavirus 16 (HPV16), a role in inhibiting the host cell autophagy has been proposed for the entire “early protein group” [9], but neither the single contribution of the viral oncogenic proteins, nor the molecular mechanisms involved in such inhibition, have been investigated. Starting from our recent results dealing with the ability of KGF/KGFR signaling in promoting autophagy [16] and with the capacity of HPV16 E5 to down-regulate the receptor expression for perturbation of epithelial homeostasis and differentiation [12, 13], we speculated that 16E5 might be the HPV16 early product major candidate for the role of interference with the autophagic process, possibly occurring through KGFR down-modulation. Consistent with this hypothesis we demonstrated, using biochemical and immunofluorence approaches, that the ectopic expression of 16E5 efficiently counteracts KGF-mediated autophagy. In fact, the inhibitory effects induced by the viral protein were comparable to those observed under receptor depletion and the forced receptor overexpression and the triggering of its signaling was able to contrast the repressive function of 16E5 on the autophagic process. These results suggest that 16E5 and KGFR would exert opposite and interplaying roles not only on epithelial differentiation, as recently proposed [13], but also on the control of autophagy.

Interestingly, taking advantage of the use of serum starvation as autophagic stimulus in which the contribution of KGFR signaling was excluded, we provided the first evidence that 16E5 affects autophagy also through transcriptional regulation. In fact, our molecular analysis showed that 16E5 is able to repress most of the autophagy core machinery genes, some of which are direct targets of p53, one of the main transcriptional inductor of the autophagic program [29]. However, differently from the 16E6 oncoprotein, whose crucial role as p53 down-regulator has been proposed [31], only a modest ability to repress p53 expression has been ascribed to 16E5 [26]. Therefore it is possible that, in the case of 16E5 expression, p53 would be mainly functionally-regulated, rather than transcriptionally-regulated. To investigate such possibility we decided to analyze also the expression of the general p53-target genes p21 and 14–3-3σ, in order to monitor p53 function in our cell model [30]. In agreement with the hypothesis of a functional regulation of p53, we found that, when 16E5 is expressed and autophagy is induced by serum starvation or KGF stimulation a significant decrease in p53-target gene transcription was observed, indicating functional repression of p53. These results provide new elements to assume that the negative impact of 16E5 on autophagy might be also due to the ability of the viral protein to induce a functional inhibition of p53 activity, which in turn results in down-regulation of autophagy genes. Moreover, the observed repressing effect on autophagy genes, which are not directly regulated by p53, suggests that 16E5 may in parallel interfere with other autophagy transcriptional regulators still unknown.

It has been reported that autophagy is linked to epithelial cell differentiation [32–34] and we have recently proposed the existence of a direct interplay between the two processes in human keratinocytes demonstrating that the induction of autophagy in response to KGFR activation is necessary for the triggering of early differentiation [16]. Accordingly with the knowledge that 16E5 acts during HPV infection perturbing keratinocyte differentiation [35, 36] and that this occurs through KGFR down-modulation [13], our present study shows that a finely controlled impairment of the autophagic process, also through KGFR down-regulation, could be one of the molecular mechanisms used by 16E5 to inhibit and delay epithelial cell differentiation for maintenance of an undifferentiated status indispensable for virus replication.

On the other hand, a close interplay between p53 activity and epidermal cell differentiation has been also proposed: in fact, in suprabasal differentiating keratinocytes, p53 is activated by the dramatic decrease of its functional repressor ΔNp63α [37] and several keratinocyte differentiation-specific markers, including Notch1, Hsp70 and keratin 14, are finely regulated by the ΔNp63α/p53 inverse functional cooperation [38–42]. Moreover, it has been observed that p53 activity promotes differentiation in HaCaT cells [43]. Based on these evidences, our results may indicate that 16E5 is able to utilize parallel and not interconnected mechanisms, involving both KGFR down-regulation and functional repression of p53, for interference with keratinocyte early differentiation and for impairment of autophagy. Since we demonstrated here that also the autophagy induced by KGF signaling appears to be transcriptionally controlled, although in a p53-independent manner, we might conclude that a transcriptional crosstalk among 16E5 and KGFR is the crucial molecular driver of epithelial deregulation during early steps of HPV infection and transformation.

## MATERIALS AND METHODS

### Cells and treatments

The human keratinocyte cell line HaCaT [17] was cultured in Dulbecco's DMEM, supplemented with 10% fetal bovine serum (FBS) plus antibiotics. HaCaT cells stably transfected with the construct pMSG 16E5 (HaCaT pMSG E5) or with the empty vector (HaCaT pMSG) [22] were cultured as reported above and were treated with 1 μM dexamethasone (Dex) for 12 h and 24 h to induce 16E5 expression. The human cervical keratinocyte cell line W12 initiated from a low-grade cervical lesion [19], which retains ~100 to 200 copies of the HPV16 episomes per cell [19, 44, 45], was cultured as previously described [19] and used at the passage 6 (W12p6). Primary cultures of human keratinocytes derived from healthy skin (HKs) were obtained from patients attending the Dermatology Unit of the Sant'Andrea Hospital of Rome; all patients were extensively informed and their consent for the investigation was given and collected in written form in accordance with guidelines approved by the management of the Sant'Andrea Hospital. Primary keratinocytes were isolated and cultured as previously described [46].

Cells were transiently transfected or cotransfected with pCI-neo expression vector containing 16E5-HA [18] (HaCaT E5, HKs E5), human KGFRwt (HaCaT KGFRwt), a kinase negative mutant KGFRY656F/Y657F (HaCaT KGFRkin-), the empty vector (HaCaT pCI-neo, HKs pCI-neo), with the pEGFP-C2 expression vector containing LC3 (engineered by Dr. Fimia, National Institute for Infectious Diseases IRCCS ‘L. Spallanzani’, Rome, Italy; and kindly provided by Prof. Francesco Cecconi, Tor Vergata University of Rome, Italy) (HaCaT EGFP-LC3, HKs EGFP-LC3, W12 EGFP-LC3) or with the pDest-mCherry-EGFP tandem expression vector containing LC3 (HaCaT mCherry-EGFP-LC3) [20]. For all transfections jetPEI^TM^ DNA Transfection Reagent (Polyplus-transfection, New York, NY, USA) or Fugene HD (Promega, Madison, WI, USA) were used according to manufacturer's instructions.

For RNA interference and *FGFR2* or *16E5* silencing, HaCaT cells were transfected with Bek small interfering RNA (FGFR2 siRNA) (Santa Cruz Biotechnology Inc., Santa Cruz, CA, USA), or with an unrelated siRNA as a control (control siRNA), while W12p6 cells were transfected with the E5 siRNA sequence (5′-TGGTATTACTATTGTGGATAA-3′) [47] or the control sequence (5′-AATTCTCCGAACGTGTCACGT-3′) [47] (Qiagen, Valencia, CA, USA), using Lipofectamine 2000 Transfection Reagent (Invitrogen, Carlsbad, CA, USA) according to the manufacturer's protocol.

For growth factor stimulation, cells were serum starved or incubated with 100 ng/ml KGF (Upstate Biotechnology, Lake Placid, NY, USA) for 24 h at 37°C.

To inhibit the autophagic degradation, cells were incubated with 20 μM leupeptin (Sigma-Aldrich Inc., Saint Louis, MO, USA) for 24 h.

### Immunofluorescence

Cells transfected with EGFP-LC3 or cotransfected with EGFP-LC3 and pCI-neo E5-HA or pCI-neo empty vector were grown on coverslips and treated or not with KGF as above, fixed with 4% paraformaldehyde in PBS for 30 minutes at 25°C followed by treatment with 0.1 M glycine for 20 minutes at 25°C and with 0.1% Triton X-100 for additional 5 minutes at 25°C to allow permeabilization. Cells were then incubated for 1 h at 25°C with mouse monoclonal anti-HA (1:50 in PBS; Covance, Berkeley, CA, USA) and the primary antibody was visualized using goat anti-mouse IgG-Texas Red (1:200 in PBS; Jackson Immunoresearch Laboratories, West Grove, PA, USA) for 30 minutes at 25°C. Nuclei were stained with DAPI (1:1000 in PBS; Sigma-Aldrich Inc.). Coverslips were finally mounted with mowiol (Sigma Aldrich Inc.) for observation. Fluorescence signals were analyzed by scanning cells in a series of sequential sections with an ApoTome System (Zeiss, Oberkochen, Germany); image analysis was performed by the Axiovision software (Zeiss) and 3D reconstruction of a selection of three central optical sections was shown in each figure. Quantitative analysis of EGFP-LC3-positive dots per cell was performed analyzing 100 cells for each sample in 5 different microscopy fields from 3 different experiments. Results have been expressed as mean values ± standard errors (SE). *p* values were calculated using Student's *t* test and significance level has been defined as *p* < 0.05.

### Western blot analysis

Cells were lysed, total protein were resolved by SDS-PAGE and transferred to reinforced nitrocellulose as previously described [16]. The membranes were blocked with 5% non fat dry milk in PBS 0.1% Tween 20 or with 3% BSA in PBS 0.1% Tween 20, and incubated with anti-Bek polyclonal antibodies (C-17, Santa Cruz Biotechnology Inc.), anti-LC3 polyclonal antibodies (MBL, Woburn, MA, USA), anti-SQSTM1 monoclonal antibody (BD Bioscience, San Josè, CA, USA) or anti-HA monoclonal antibody (Covance) followed by enhanced chemiluminescence detection (ECL, Amersham, Alington Heights, IL, USA). The membranes were rehydrated and probed again with anti-β actin (Sigma Aldrich Inc.) monoclonal antibody, for equal loading. Densitometric analysis was performed using Quantity One Program (Bio-Rad Laboratories, Hercules, CA, USA). The resulting values from three different experiments were normalized, expressed as fold increase respect to the control value and reported in graph as mean values ± standard deviation (SD). Student's *t* test was performed and significance levels have been defined as *p* < 0.05.

### Transmission electron microscopy

HaCaT pMSG E5 and HaCaT pMSG cells treated with Dex and stimulated with KGF for 24 h as above were washed three times in PBS and fixed with 2% glutaraldehyde in PBS for 2 h at 4 °C. Samples were postfixed with 1% osmium tetroxide in veronal acetate buffer (pH 7.4) for 1 h at 25 °C, stained with uranyl acetate (5 mg/ml) for 1 h at 25 °C, dehydrated in acetone and embedded in Epon 812 (EMbed 812, Electron Microscopy Science, Hatfield, PA, USA). Ultrathin sections were examined unstained or poststained with uranyl acetate and lead hydroxide, under a Morgagni 268D transmission electron microscope (FEI, Hillsboro, OR, USA) equipped with a Mega View II charge-coupled device camera (SIS, Soft Imaging System GmbH, Munster, Germany) and analyzed with AnalySIS software (SIS).

### Primers

Oligonucleotide primers for target genes and for the housekeeping gene were chosen with the assistance of the Oligo 5.0 computer program (National Biosciences, Plymouth, MN, USA) and purchased from Invitrogen. The following primers were used: for *HPV 16E5* target gene 5′-CGCTGCTT TTGTCTGTGTCT-3′(sense), 5′-GCGTGCATG TGTATGTATTAAAAA-3′(antisense); for *BECN1* target gene 5′-GGATGGTGTCTCTC GCAGAT-3′(sense), 5′-TTGGCACTTTCTGTGG ACAT-3′(antisense); for *ATG5* target gene5′-CAACTTGTTTCACGCTATATCAGG-3′(sense), 5′-CACTTTGTCAGTTACCAACGTCA-3′(antisense); for *ATG7* target gene 5′-CCGTGGAATTGAT GGTATCTG-3′(sense), 5′-TCATCCGATCGTCACTGCT-3′(antisense); for *MAP1LC3B* target gene 5′-CGCACC TTCGAACAAAGAG-3′(sense), 5′-CTCACCCTTG TATCGTTCTATTATCA-3′ (antisense); for *ULK1* target gene 5′-CAGACGACTTCGTCATGGTC-3′(sense), 5′-AGCTCCCACTGCACATCAG-3′(antisense); for *ULK2* target gene 5′-TTTAAATACAGAACGACCAATGGA-3′(sense), 5′-GGAGGTGCCAGAACACCA-3′(antisense); for *ATG4a* target gene 5′-CCGTCCGTAGTCAAGT TGC-3′(sense), 5′-TCTGATCTTCATACTTGGATAAAA CTG-3′ (antisense); for *p21* target gene: 5′-TCACTGTCTTGTACCCTTGTGC-3′(sense), 5′-GGC GTTTGGAGTGGTAGAAA-3′(antisense); for *14–3-3 sigma* target gene: 5′-GACACAGAGTCCGGCATTG–3′(sense), 5′-ATGGCTCTGGGGACACAC-3′(antisense); for the housekeeping 18S rRNA gene: 5′-AACCAACCCGGTCAGCCCCT-3′(sense), 5′-TTC GAATGGGTCGTCGCCGC-3′(antisense). For each primer pair, we performed no-template control and no-reverse-transcriptase control (RT negative) assays, which produced negligible signals.

### RNA extraction and cDNA synthesis

RNA was extracted using the TRIzol method (Invitrogen) according to manufacturer's instructions and eluted with 0, 1% diethylpyrocarbonate (DEPC)-treated water. Each sample was treated with DNAase I (Invitrogen). Total RNA concentration was quantitated by spectrophotometry. 1 μg of total RNA was used to reverse transcription using iScript^TM^ cDNA synthesis kit (Bio-Rad Laboratories) according to manufacturer's instructions.

### PCR amplification and real-time quantitation

Real-time PCR was performed using the iCycler Real-Time Detection System (iQ5 Bio-Rad) with optimized PCR conditions. The reaction was carried out using iQ SYBR Green Supermix (Bio-Rad Laboratories) and the thermal cycling program was performed as previously described [12]. Real-time quantitation was performed with the help of the iCycler IQ optical system software version 3.0a (Bio-Rad Laboratories), according to the manufacturer's manual. Results are reported as mean ± standard error (SE) from three different experiments in triplicate.

## SUPPLEMENTARY FIGURE


