# Supplementary material for: HPV16 E5 deregulates the autophagic process in human keratinocytes
Source: Oncotarget. 2015 Mar 19;6(11):9370–86. doi: 10.18632/oncotarget.3326 (PMC4496223; doi:10.18632/oncotarget.3326)
Supplement: Supplementary file 1 [file oncotarget-06-9370-s001.pdf]

SUPPLEMENTARY FIGURE

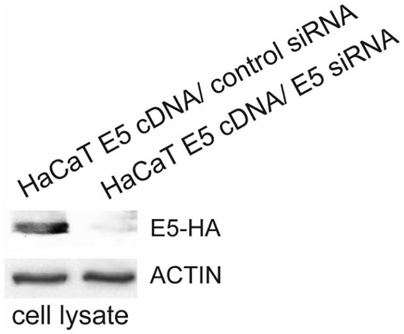

Supplementary Figure 1: E5 siRNA induce an efficient depletion of 16E5 protein in transiently transfected HaCaT E5 cells. HaCaT cells were doubly transfected with pCI-neo E5-HA cDNA and E5 siRNA or control unrelated siRNA. Western blot analysis using anti-HA monoclonal antibody shows that the band at the molecular weight corresponding to 16E5 protein is decreased in HaCaT E5 cDNA/E5siRNA as expected.
